# Dynamic visualization of extracellular matrix components in *S. aureus* colony biofilms reveals functional amyloids leading to the formation of cap-like structures

**DOI:** 10.1016/j.bioflm.2025.100318

**Published:** 2025-09-18

**Authors:** Tianqi Zhang, Julian Bär, Lovisa Risberg, Alejandro Gómez Mejia, Hugo Hammar, Susanne Löffler, Daniel Erik Otzen, Maria Andreasen, Rikke Louise Meyer, Keira Melican, Annelies S. Zinkernagel, Agneta Richter-Dahlfors

**Affiliations:** a AIMES – Center for the Advancement of Integrated Medical and Engineering Sciences, Karolinska Institutet and KTH Royal Institute of Technology, SE-171 77, Stockholm, Sweden; b Department of Neuroscience, Karolinska Institutet, SE-171 77, Stockholm, Sweden; c Department of Infectious Diseases and Hospital Epidemiology, University Hospital Zurich, University of Zurich, Zurich, Switzerland; d Interdisciplinary Nanoscience Centre (iNANO) and Department of Molecular Biology and Genetics, Aarhus University, Aarhus, 8000, Denmark; e Department of Biomedicine, Aarhus University, Aarhus, 8000, Denmark

## Abstract

*Staphylococcus aureus* infections represent a clinical challenge due to their propensity to form biofilms and the increasing prevalence of antibiotic resistance. The ability of *S. aureus* to form biofilm affects clinical outcome, but techniques to study extracellular matrix (ECM) in *S. aureus* biofilms are lacking. Here, we present an agar-based method in which the optotracer EbbaBiolight 680 (Ebba680) is used to visualize ECM formation alongside evaluation of colony growth dynamics in agar colonies. As models for colony biofilms, we use drop inoculation for macrocolony formation or spread-plating for single-cell derived colonies. Kinetic fluorescence spectroscopy combined with time-lapse microscopy showed bright fluorescence signals, revealing different spatial-temporal appearance of ECM in macrocolonies versus single-cell derived colonies. In contrast, the microstructure was conserved between the two types of colonies. Detailed characterization of the biofilm microstructures by confocal microscopy revealed Ebba680 binding targets interspersed between cells as well as in a cap-like structure formed on the outer surface of the biofilm. Accessory gene regulator (*agr*) controlled expression of Ebba680 binding target(s) and the binding of Ebba680 to synthetic fibrillated phenol soluble modulins (fPSMs) suggests these functional amyloids act as targets for Ebba680 in the biofilm ECM. By upgrading ColTapp, an application developed for colony radius quantification, to also analyze fluorescence images, concurrent analysis of Ebba680-stained ECM and colony growth was achieved. This provided a new dimension to the assessment of colony biofilms. Detailed phenotypic characterization of clinical isolates is critical for treatment decision making, and enhanced screening which includes ECM as presented here has potential to facilitate treatment decisions in problematic staphylococcal infections.

## Highlights

• *S. aureus* biofilm ECM production can be tracked in real-time by EbbaBiolight 680.
• ECM forms a cap structure on the outer surface of *S. aureus* biofilm colonies.
• Fibrillated phenol soluble modulins are a key binding target for EbbaBiolight 680.
• Kinetics of ECM production differs in macrocolonies vs single cell derived colonies.
• Combining ECM detection with ColTapp gives a more complete colony analysis.

## 1 Introduction

*Staphylococcus aureus* (*S. aureus*) is a major human pathogen which colonizes human skin and mucous membranes [1,2]. As a coloniser, *S. aureus* is not typically problematic but once it invades underlying tissue via injuries or indwelling devices, it can cause a broad range of clinical manifestations [3]. The ability of *S. aureus* strains to form biofilm [4,5] renders treatment challenging and facilitates the emergence of antibiotic resistance [[6], [7], [8]]. Bacterial biofilms are multicellular communities encased in an extracellular matrix (ECM) and their intricate structure enables bacteria to withstand environmental stresses, including antibiotic treatment and host immune defenses [[9], [10], [11], [12]]. ECM composition varies greatly in different bacteria and growth conditions, but the general building blocks are exopolysaccharides, proteins, nucleic acids, lipids and biosurfactants [8,13]. A widespread mechanism that provides structural stability to biofilms is the formation of extracellular functional amyloids from amyloidogenic proteins and peptides [14,15]. In contrast to pathogenic amyloids, which are often associated with neurodegenerative diseases, the conformation of functional amyloids serves a biological purpose. This includes conferring structural stability, storage of peptides or hormones, sequestration of pro-inflammatory factors, protection, enzymatic activity and virulence [[14], [15], [16]]. The most well-described bacterial functional amyloids in biofilm ECM are the curli fibers, identified in Gram-negative bacteria Salmonella and *Escherichia coli* and abundant in environmental Proteobacteria and Bacteriodetes [17,18]. Another example is Fap fibrils found in different Proteobacteria including *Pseudomonas* [[19], [20], [21]], while bacterial amyloids have also been described in *Bacillus subtilis* (TasA) and other species [14,15].

In *S. aureus,* functional amyloids composed of phenol soluble modulins (PSMs) have been described as part of the structural scaffold of biofilms [[22], [23], [24]]. The expression of PSMs is controlled by quorum sensing via the accessory gene regulator (*agr*) [25,26]. In monomeric forms, the shorter PSMα1-4 and longer PSMβ1-2 peptides display an amphipathic α-helical structure and regulate biofilm maturation and dissemination [22,[27], [28], [29]]. As they assemble into amyloids, most PSM fibrils adopt a cross-β structure, except for PSMα3 that forms a unique cross-α fibrillar structure [27,30,31]. The fibrils function as structural scaffold in the biofilm matrix – sometimes in a complex with DNA, and enhance antibiotic resistance by barrier function, drug adsorption and direct enzymatic activity towards β-lactams [32]. Studying functional amyloids in the ECM of Staphylococci is critical to understand their role in biofilm-related infections and their possible contribution to the emergence of antibiotic resistance. A lack of tools that allow the study of real-time development of biofilm in a controlled and non-interventional manner has, however, hampered the progress in this field.

Over the last decade we have developed optotracing, which allows the visualization and identification of amyloids and glucans in bacterial biofilm formation [[33], [34], [35]]. Optotracers are small molecules which change their photophysical properties upon target binding. This leads to an on-switch of fluorescence and allows identification of specific targets based on the spectral fingerprints obtained from the excitation and emission spectra of bound optotracer [34,36]. The optotracer EbbaBiolight has been used in various models to reveal the role of curli in Salmonella biofilms and the effect of electrically charged surfaces on bacterial growth and ECM production [[37], [38], [39]]. In *E. coli*, EbbaBiolight has shed new light on the role of ECM and biofilm formation in a proximal-tubule-on-a-chip model [40], and has been used to assess antibacterial wound dressings [41] and responses to antimicrobial peptides [42]. In other Gram-negative bacteria such as *Burkholderia cenocepacia*, *Klebsiella pneumoniae* and *Pseudomonas aeruginosa*, EbbaBiolight was used as a general ECM stain [[43], [44], [45], [46], [47]], while in *Candida albicans*, the spectral fingerprint of the optotracer has been shown to differentiate between planktonic yeast cells and biofilm hyphae [39]. To increase the relevance of this technique for clinical microbiology, we developed an optotracer based agar model in which biofilm formation is studied in bacterial colonies grown on agar supplemented with EbbaBiolight 680 (Ebba680). Although the physiology and structure of colony biofilms differ from submerged biofilms [48,49], colonies are recognized as biofilm-like communities that share key features in terms of ECM production, spatial stratification, and phenotypic heterogeneity [50,51]. Importantly, macrocolonies generated by drop inoculation on agar surfaces are a widely established experimental model for studying biofilm formation in Gram-negative species [52,53]. We have used this model successfully to reveal the kinetic development of the intricate architecture of colony biofilms in Salmonella where Ebba680 specifically bound the amyloid curli [54] and in an antibiotic susceptibility test (AST) targeting the Salmonella biofilm lifestyle [37]. In uropathogenic *E. coli* (UPEC), our model allowed for visualization of the spatiotemporal localization of amyloid curli and bacterially produced cellulose during biofilm formation with unprecedented resolution [55].

Here, we further develop our agar-based model to study colony biofilm formation and growth dynamics in Gram-positive *S. aureus*. As *S. aureus* is notorious for strain heterogeneity, particularly regarding biofilm formation [[56], [57], [58]], we use a selection of strains to represent a range of phenotypes. For the method development we use SH1000, a stable, well characterised methicillin sensitive (MSSA) strain. SH1000 was constructed by repairing the *rsbU* gene in 8325-4, thereby restoring the σB activity and the reliable production of biofilm-associated factors [[23], [59]]. This, along with its genetic tractability makes SH1000 a good choice for studying biofilms in *S. aureus*. We also use JE2, a derivative of LAC and representative of the USA300 methicillin resistant (MRSA) lineage [60,61], as well as the clinical isolate Cl1149 [62,63] as representatives of other major S*. aureus* phenotypes. Tracking drop-inoculated macrocolonies and single cell derived colonies, we find that Ebba680 fluorescence appears with a marked delay compared to colony growth. Using confocal microscopy, we determine that Ebba680 fluorescence originates from extracellular components and that Ebba680 binding targets form a cap-like structure on mature *S. aureus* colonies. We associate the Ebba680 binding signal to synthetic fibrillated PSMs, indicating their potential localization at the outer surface of the colony. We further demonstrate how tracking extracellular components can complement tracking of colony growth dynamics and aid in characterization of biofilm formation in *S. aureus*.

## 2 Results

### 2.1 Growth kinetics of *S. aureus* macrocolonies reveal delayed appearance of Ebba680 fluorescence

**Fig. 1:**
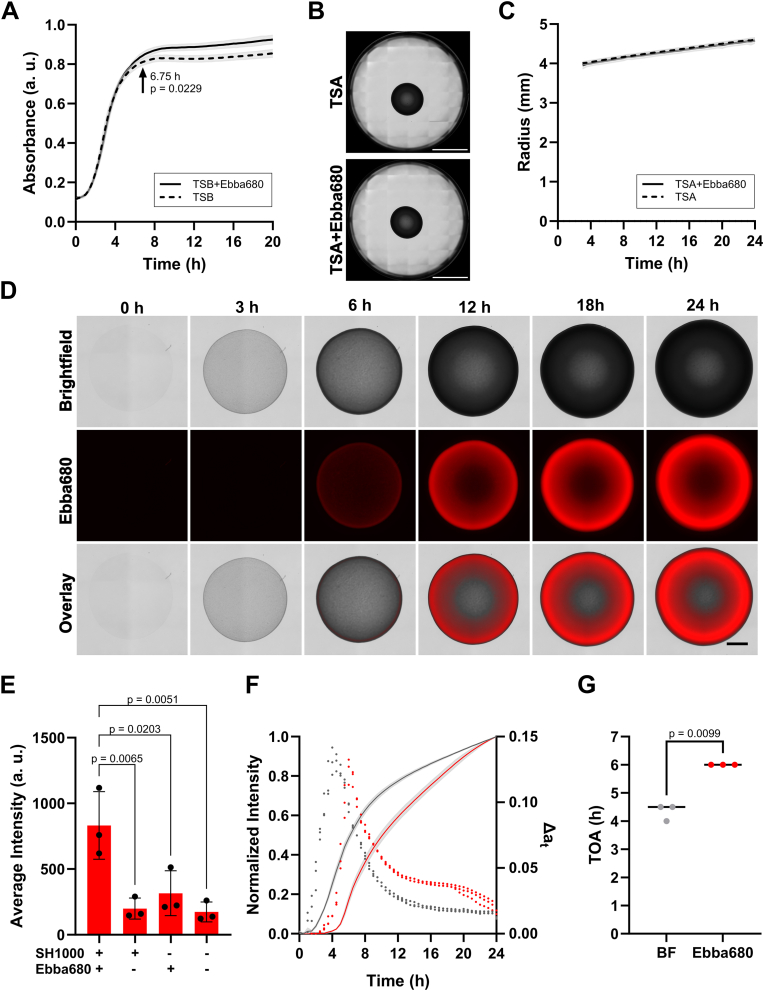
**Real-time visualization of SH1000 macrocolonies. A)** Absorbance at 600 nm shows growth of SH1000 in TSB + Ebba680 and TSB (n = 3). Values are displayed as mean values ± SD (shading). A two-way Anova with Šídák's multiple comparisons was used to compare the mean values for each timepoint. The arrow indicates the time point when the curves start to diverge significantly. **B)** Brightfield images of SH1000 macrocolonies grown for 24 h on TSA or TSA + Ebba680 in the 6-well plate agar biofilm assay (n = 3). Scale = 10 mm. **C)** Radius of SH1000 macrocolonies growing on TSA + Ebba680 (red) and TSA (grey) between 3 and 24 h (n = 3). Values are displayed as mean values ± SD (shading). A two-way Anova with Šídák's multiple comparisons was used to compare the mean values for each timepoint and no divergence was found (p > 0.9999). **D)** Representative images of SH1000 macrocolonies on TSA + Ebba680 (n = 3). Upper panel: Brightfield, central panel: Ebba680 fluorescence, lower panel: Overlay of Brightfield and Ebba680 fluorescence. Scale = 2 mm. **E)** Average intensity of red fluorescence in SH1000 macrocolonies grown for 24 h (n = 3) determined from averaging all areas read by the plate reader in area scan mode. Statistical testing was performed using a one-way ANOVA with Tukey's multiple comparisons test. Non-significant comparisons (p > 0.7) are omitted. **F)** Normalized Intensity (continuous lines) obtained by Min-Max normalization of average intensity of brightfield (grey) and Ebba680 fluorescence (red) images of SH1000 macrocolonies growing on TSA + Ebba680 (n = 3). Values are displayed mean values ± SD (shading). For each replicate, the difference in consecutive values of the rolling average (Δa_t_) of the Normalized Intensity with window size 2 (dots) is plotted for brightfield (grey) and Ebba680 fluorescent (red). **G)** Time of Appearance (TOA) of brightfield (BF) and Ebba680 fluorescence obtained from the time point of Δa_t_ reaching its maximum. Significance was determined by a paired *t*-test. (For interpretation of the references to colour in this figure legend, the reader is referred to the Web version of this article.)

We first investigated if Ebba680 influenced the growth of *S. aureus.* As growth curves of *S. aureus* SH1000 in liquid cultures with and without Ebba680 largely overlapped (Fig. 1A), we continued to grow macrocolonies on Ebba680 supplemented agar. The radii of macrocolonies formed on tryptic soy agar (TSA) or on TSA supplemented with Ebba680 (TSA + Ebba680) also overlapped (Fig. 1B and C). Since this indicated that the optotracer did not affect bacterial growth, we continued to inoculate SH1000 on TSA + Ebba680 in 6 well-plates. During incubation inside a temperature-controlled automated microscope, a matrix of images covering the entire area of each well, was collected every 30 min. Analysis of the growth kinetics in the brightfield channel showed how drop inoculated SH1000 grown on TSA + Ebba680 developed into a macrocolony with increasing radius and darker appearance (Fig. 1D, Supplementary Movie 1). Ebba680 (red) fluorescence appeared at 6 h and increased in intensity throughout incubation. The highest fluorescence intensity was initially located at the periphery of the macrocolony, from where it seemed to spread towards the centre. A similar growth pattern was observed in brightfield for SH1000 growing on TSA, but as expected, the macrocolonies lacked red fluorescence (Supplementary Movie 2). The end-stage fluorescence intensity, obtained from a plate reader in area scan mode, confirmed a strong signal from the macrocolony growing on TSA + Ebba680, and that background fluorescence of TSA without Ebba680 or uninoculated TSA + Ebba680 was low (Fig. 1E). Normalized intensity of brightness and Ebba680 fluorescence from timelapse images showed monotonical increase (Fig. 1F). To determine the time of appearance (TOA), we used the timepoint where the difference between consecutive rolling averages calculated with window size 2 (Δa_t_) reached its maximum (Fig. 1G). The average TOA of the brightfield signal was 4.3 ± 0.3 h, while fluorescence showed TOA of 6.0 ± 0.0 h. The delay seen for the fluorescence signal suggests that Ebba680 binds to bacterially secreted biofilm component(s) rather than directly to the bacterial cells.

### 2.2 Confocal microscopy reveals a cap-like structure forming on the surface of macrocolonies

**Fig. 2:**
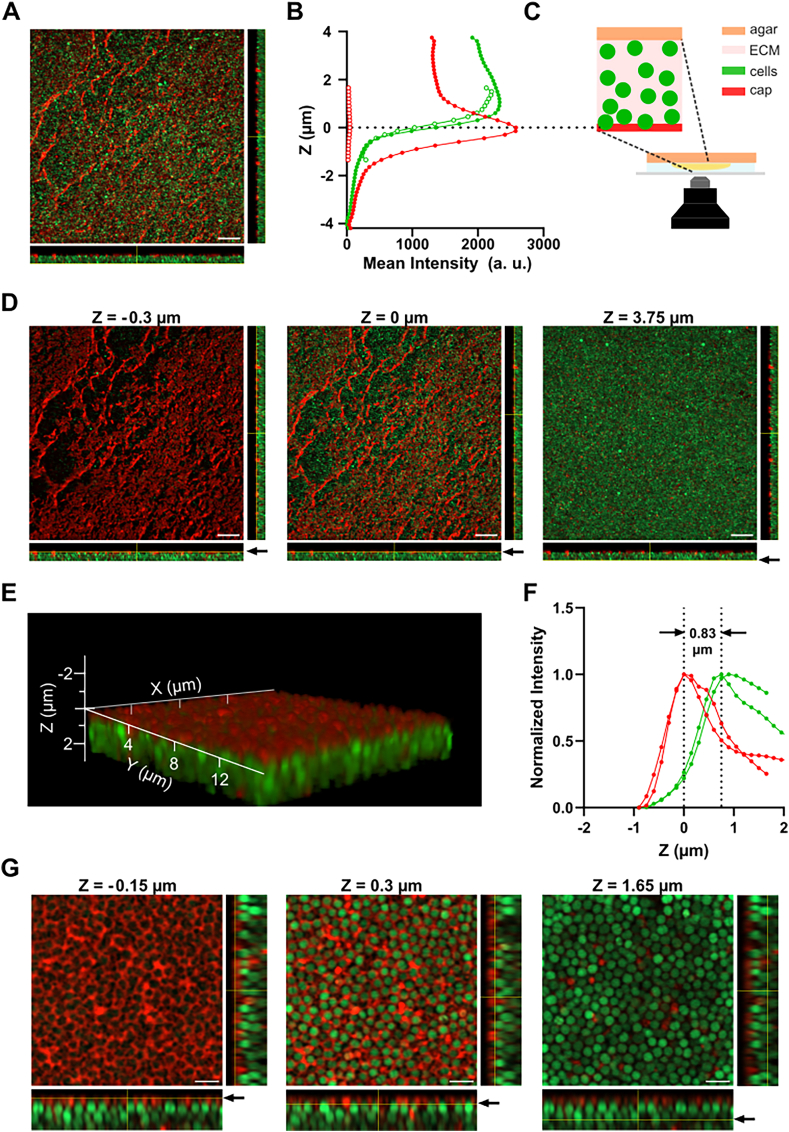
**Localization of Ebba680 signal in SH1000-GFP macrocolonies. A)** Maximum intensity projection of a confocal stack of SH1000-GFP (green) macrocolonies grown on TSA + Ebba680 (red) for 24 h. Orthogonal projections show fluorescence in x-z and y-z direction. Scale bar = 10 μm. **B)** Mean Intensity of GFP (green) and Ebba680 (red) intensity through the depth Z (μm) of the biofilm. SH1000-GFP Macrocolonies were grown on Ebba680+TSA (filled circles) or TSA (open circles) for 24 h and imaged with a 63X objective lens (n = 1). Mean Intensity of each z-slice in the red and green channel was plotted so that the brightest z-slice of the red channel was set to Z = 0 μm. The graph was rotated by 90°. **C)** Illustration, linking confocal setup with intensity plots. **D)** Representative images from indicated depths of SH1000-GFP (green) macrocolonies, grown for 24 h on TSA + Ebba680 (red) and imaged with a 63X objective lens (n = 1). The depth Z (μm) is indicated above each image, and in the orthogonal projections by yellow line and arrow. Scale bar = 2 μm. **E)** A 3D reconstruction of a representative stack showing SH1000-GFP (green) macrocolonies, grown for 24 h on TSA + Ebba680 (red) and imaged with 63X objective lens and airyscan detection. **F)** Normalized Intensity of GFP (green) and Ebba680 (red) fluorescence in each z-slice of SH1000-GFP macrocolonies grown for 24 h on TSA + Ebba680 and imaged with 63X objective lens and airyscan detection (n = 2). Min-Max Normalization was performed for each replicate in the red and green channel. The brightest z-slice of the red channel was set to Z = 0 μm for each replicate. **G)** Representative images from indicated depths of the stack shown in E. The depth Z (μm) is indicated above each image, and in the orthogonal projections by yellow line and arrow. Scale bar = 2 μm. (For interpretation of the references to colour in this figure legend, the reader is referred to the Web version of this article.)

The delayed appearance of fluorescence prompted us to investigate the spatial localization of the Ebba680 binding target. GFP-expressing SH1000 (SH1000-GFP) were grown as macrocolonies for 24 h and imaged using confocal microscopy. In macrocolonies grown on TSA + Ebba680, the maximum intensity projection showed mixed Ebba680 (red) and GFP (green) fluorescence, while orthogonal projections as well as the 3D reconstruction showed a distinct separation of signals at different depths (Fig. 2A, Supplementary Movie 3). Macrocolonies grown in the absence of Ebba680 showed only green signal (Supplementary Fig. 1). To understand the spatial relationship between red and green fluorescence through the depth (Z) of the biofilm, the mean intensity from each channel in each focal plane of the stacks was measured and shown as a graph in which the focal plane with the highest intensity of red fluorescence was aligned to Z = 0 μm (Fig. 2B). The graph was rotated 90° to match the experimental set-up we used for non-interventional biofilm imaging (Fig. 2C). The data revealed that red fluorescence extended beyond the outside of the macrocolony (Z < 0) where green fluorescence from the bacterial cells was low. In the macrocolony (Z > 0), red and green fluorescence co-existed. These data were confirmed by images of selected focal planes, showing red fluorescence dominating the top section of the macrocolonies (Fig. 2D). A few focal planes below, mixed red and green fluorescence were observed, while green fluorescence was most prominent at the bottom near the agar. The 3D reconstruction showed a carpet-like structure of bacterial cells with red fluorescence primarily localised on top (Supplementary Fig. 2).

To determine if the Ebba680 signal originated from an extracellular location, we examined macrocolonies at single cell resolution using confocal imaging with airyscan detection, which in addition to increased optical resolution provides enhanced signal to noise ratio. After growth for 3 h, no extracellular Ebba680 fluorescence was observed in macrocolonies analysed at standard settings (Supplementary Fig. 3A, Supplementary Movie 4), however, cell envelopes showed red fluorescence when brightness was increased 7x (Supplementary Fig. 4A and B). This confirmed findings from previous publications [64,65]. At 6 h, red fluorescence had appeared and was present throughout the macrocolony (Supplementary Fig. 3B, Supplementary Movie 5). From here onwards, production of the Ebba680 binding target(s) continued, and at 24 h, a 3D reconstruction of the macrocolony showed a carpet-like assembly of green cells with a top layer of red fluorescence (Fig. 2E, Supplementary Movie 6). The normalized mean fluorescence intensity from focal planes throughout the depth of the macrocolony revealed unimodal, bell-shaped curves for both channels (Fig. 2F). The maximum of the red curve was shifted 0.83 ± 0.11 μm (n = 2) towards the outside of the colony compared to the maximum of the green curve. Selected focal planes confirmed that an outer layer of red fluorescence had formed (Fig. 2G, Supplementary Fig. 4). Just below, red patches of extracellular material were interspersed between cells. At the bottom of the macrocolony, green fluorescence dominated. Taken together, this shows that the high-intensity Ebba680 binding target(s) are extracellular components secreted by bacteria as they form a macrocolony on agar.

### 2.3 Extracellular component production in single cell derived colonies

**Fig. 3:**
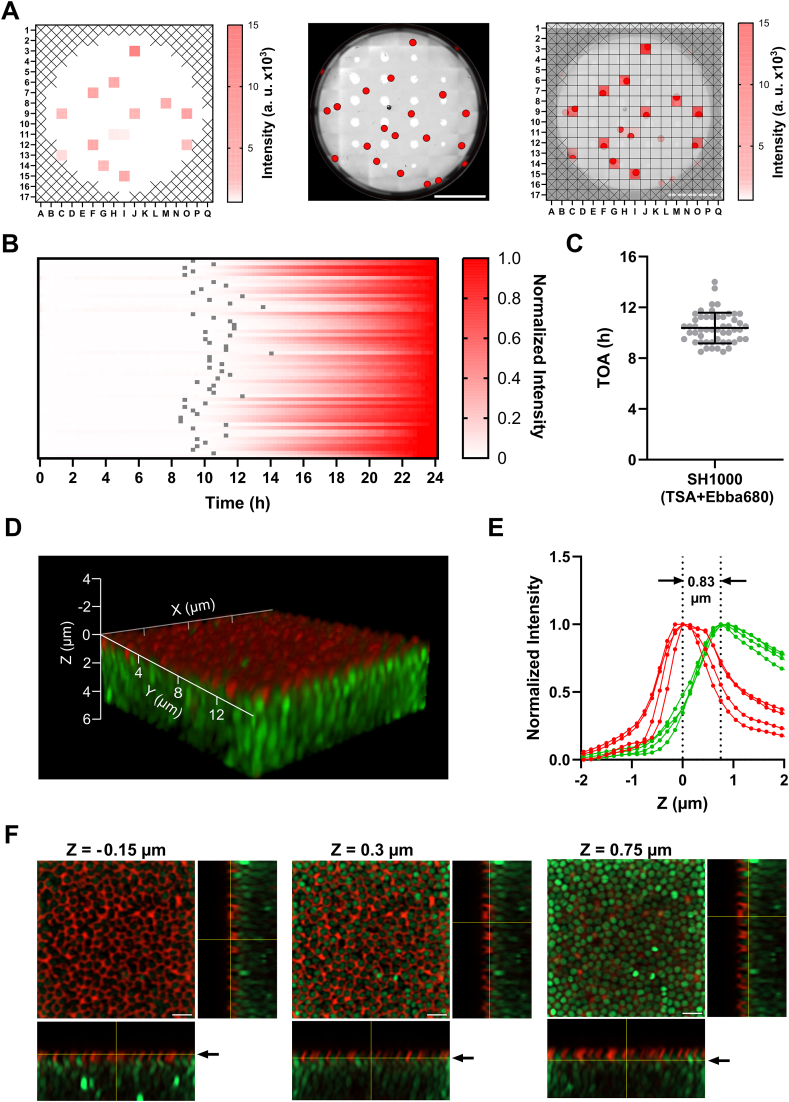
**Tracking ECM production in single cell derived colonies of SH1000. A)** Left: Representative heatmap showing Ebba680 fluorescence of SH1000 single cell derived colonies, grown on TSA + Ebba680 after 24 h. Middle: The same well imaged in the automated microscope shown as an overlay of brightfield (grey) and Ebba680 fluorescence (red). Scale bar = 10 mm. The regular white dots are reflections caused by optical artefacts. Right: Overlay of heatmap from area scan and microscope image to illustrate some colonies appearing on the edge or in-between areas. **B)** Heatmap showing Min-Max normalized Ebba680 fluorescence from 56 colonies within n = 3 experiments of SH1000 grown on TSA + Ebba680 obtained by area scan (one line per colony). Each horizontal line represents one colony. Grey markers show the time of appearance (TOA) where (Δa_t_) with window size 7 was larger than the threshold (Δa_t_ = 0.01 h) and TOA >2 h (52 colonies). **C)** TOA from 52 colonies with Mean value ± SD. **D)** A 3D reconstruction of a representative stack showing SH1000-GFP (green) single cell derived colonies, grown for 24 h on TSA + Ebba680 (red) and imaged with 63X objective lens and airyscan detection. **E)** Normalized Intensity of GFP (green) and Ebba680 (red) fluorescence in each z-slice of SH1000-GFP single cell derived colonies grown for 24 h on TSA + Ebba680 and imaged with 63X objective lens and airyscan detection (n = 4). Min-Max Normalization was performed for each replicate in the red and green channel. The brightest z-slice of the red channel was set to Z = 0 μm for each replicate. **F)** Representative images from indicated depths of the stack shown in D. The depth Z (μm) is indicated above each image, and in the orthogonal projections by yellow line and arrow. Scale bar = 2 μm. (For interpretation of the references to colour in this figure legend, the reader is referred to the Web version of this article.)

There is a clinical incentive to perform biofilm analysis based on single cell derived colonies, as this may allow detection of phenotypic heterogeneity in clinical isolates. To develop this assay, we spread-plated SH1000 onto the agar of 6-well plates and recorded the development of Ebba680 fluorescence intensity using the area scan mode of a plate reader during growth for 24 h. By showing the 24 h end values as a heatmap, a pattern of 177 squares emerged, representing the intensities of Ebba680 fluorescence at corresponding sites on the agar (Fig. 3A). An overview image of the well generated by overlaying the brightfield and Ebba680 fluorescence channels in an automated microscope, identified the location of each colony. By matching the spectrophotometric data with the image, we confirmed that the area scan method can be applied to track the dynamics of single cell derived colony growth. As colonies are randomly distributed on the surface of the agar and may be located between two reading areas, intensity reading from the area scan mode are subject to a degree of variance. To account for this, the normalized intensity of 56 tracked colonies from multiple experiments was used to determine the time of appearance (TOA) of the colonies (Fig. 3B). This revealed an average TOA of 10.4 ± 1.2 h (Fig. 3C), which is considerably longer than the 6 h TOA of Ebba680 appearance in macrocolonies (Supplementary Table 2). This reflects the different kinetics of biofilm formation using single bacteria inoculum versus macrocolonies.

To investigate the biofilm microstructure of single cell derived colonies, we performed confocal imaging with airyscan detection of SH1000-GFP colonies (Supplementary Fig. 6). The 3D reconstruction showed green cells assembled in a carpet-like structure with a top layer of red fluorescence (Fig. 3D, Supplementary Movie 7). The normalized mean fluorescence intensity from focal planes throughout the depth of the single cell derived colony showed unimodal, bell-shaped curves for both channels with a shift of 0.83 ± 0.09 μm (n = 4) (Fig. 3E). Analysis of selected focal planes confirmed the formation of an outer, red fluorescent layer containing Ebba680 binding target(s) (Fig. 3F). Since these data reflect our observations from macrocolonies in Fig. 2D–F, we conclude that the microstructure adopted by the biofilm is independent of the method for inoculation.

### 2.4 Fibrillated PSMs as Ebba680 binding targets

**Fig. 4:**
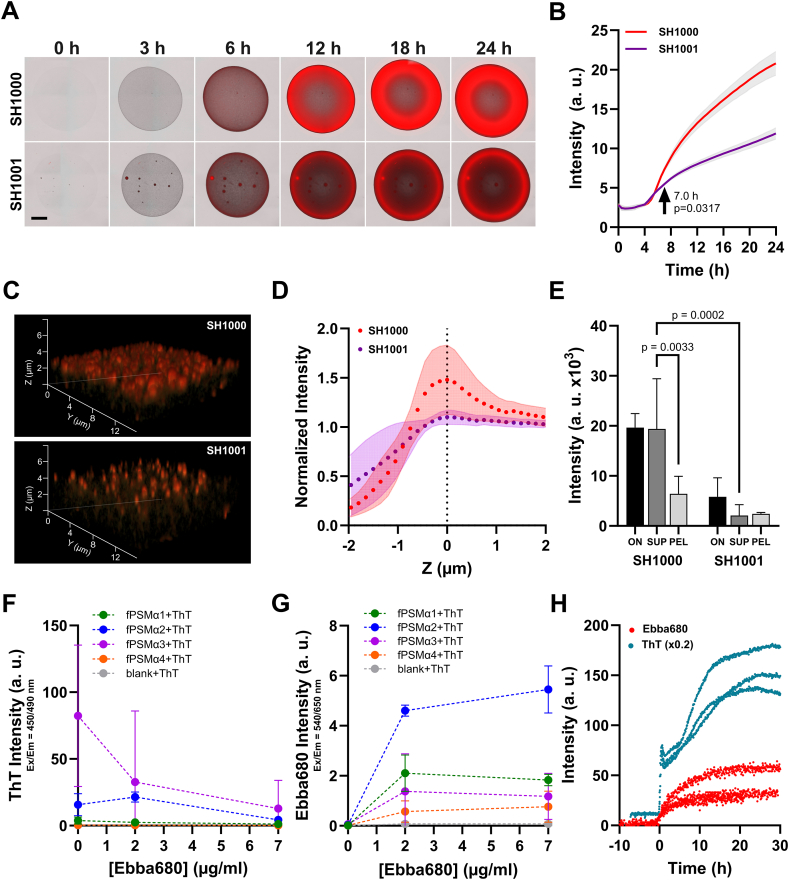
**Extracellular localization of the binding target of Ebba680. A)** Representative images with overlay of brightfield (grey) and Ebba680 (red) channels show SH1000 (upper panel) and SH1001 (lower panel) macrocolonies grown on TSA + Ebba680 (n = 3). Scale bar = 2 mm. **B)** Ebba680 fluorescence intensity from SH1000 and SH1001 macrocolonies growing on TSA + Ebba680 (n = 3). A two-way Anova with Šídák's multiple comparisons was used to compare the mean values for each timepoint. The arrow indicates the time point when the curves start to diverge significantly. **C)** 3D reconstructions of representative airyscan stacks of SH1000 (upper) and SH1001 (lower) single cell derived colonies grown for 24 h on TSA + Ebba680 (red). **D)** Normalized Intensity of Ebba680 fluorescence (mean value ± SD (shading)) in each z-slice when SH1000 and SH1001 single cell derived (n = 3) and macrocolonies (n = 3), grown for 24 h on TSA + Ebba680 were imaged with 63X objective lens and airyscan detection. For each replicate, Min-Max Normalization was performed, and the brightest z-slice of the red channel was set to Z = 0 μm. **E**) Ebba680 fluorescence intensity from SH1000 and SH1001 in overnight cultures (ON), supernatants (SUP) and pellets (PEL), baseline subtracted with fluorescence intensity from blank (n = 3). A Two-way Anova with Šídák's multiple comparisons test was performed to determine if the differences between the mean values in the two indicated groups were significant. **F)** ThT Intensity measured at λ_Ex_ = 450 nm and λ_Em_ = 490 ± 10 nm showing displacement of ThT binding to fPSMα1-4 at 0, 2, and 7 μg/mL Ebba680. An insert shows y-axis with increased resolution at lower intensities. **G)** Ebba680 intensity measured at λ_Ex_ = 540 nm and λ_Em_ = 650 ± 10 nm shows Ebba680 binding at 0, 2 and 7 μg/mL to fPSMα1-4 pre-stained with ThT. **H)** Fibrillation kinetics of PSMβ1 in presence of 2 μg/mL Ebba680 and 64 μg/mL ThT (n = 3). Baseline fluorescence from PSMβ1 was subtracted from each replicate and the timepoint when Ebba680 ≥ 10 a. u. and ThT ≥100 was set to t = 0 h for each replicate. For visualization, ThT intensity was scaled by a factor of 0.2. (For interpretation of the references to colour in this figure legend, the reader is referred to the Web version of this article.)

While our experiments demonstrated Ebba680 as a marker for extracellular components in real-time analysis of *S. aureus* colony dynamics, the nature of the binding target was still unknown. Previous identification of the functional amyloid protein curli as binding target for Ebba680 in Salmonella and *E. coli* biofilms [[37], [54], [55]] lead us to investigate whether fibrillated PSMs secreted by *S. aureus* may represent binding targets for Ebba680. Since PSM transcription requires the *agr* system, we first compared the wildtype SH1000 (wt) and the *Δagr* mutant SH1001 (*Δagr*), which has previously been shown to lack production of extracellular fibers [23]. Timelapse imaging of drop-inoculated macrocolonies on TSA + Ebba680 showed the expected appearance of strong red fluorescence from the wt, whereas in the *Δagr* mutant, red fluorescence appeared only as a thin ring at the periphery of the macrocolony (Fig. 4A, Supplementary Movie 8). No fluorescence was observed when the *Δagr* mutant grew without Ebba680 (Supplementary Movie 9). Radius analysis of wt and mutant macrocolonies on TSA and TSA + Ebba680 showed the same growth (Supplementary Fig. 7). In both strains, the fluorescence intensities over the first 7 h were similar (Fig. 4B). Then, a pronounced intensity increase occurred in the wt, which was less prominent in the *Δagr* mutant, suggesting that the *Δagr* mutant secreted less Ebba680 binding targets than the wt. To investigate the localization of red fluorescence in the two strains, we imaged single cell derived colonies by confocal microscopy with airyscan detection. 3D reconstructions of image stacks showed the typical structure of Ebba680 fluorescence in the wt, whereas the *Δagr* mutant showed a foci-like appearance (Fig. 4C). The normalized mean fluorescence intensity from focal planes throughout the depth of the wt colony showed the typical unimodal distribution of red fluorescence intensity along the z-axis with a distinct maximum (Fig. 4D). In contrast, fluorescence of the *Δagr* mutant showed a sigmoidal curve entering a plateau, indicative of colonies lacking the cap-like structure on their surfaces. Having confirmed that overnight cultures of wt and *Δagr* mutant grow to the same cell density in TSB (Supplementary Fig. 8), we investigated whether the Ebba680 binding target were secreted by analysing fluorescence signals from Ebba680 added to the supernatants and reconstituted pellets. Ebba680 intensity in the wt supernatant was equally high as in the overnight culture, whereas signals from the pellet were significantly reduced (Fig. 4E). This confirmed that in the wt, a major fraction of the Ebba680 target is secreted. In the *Δagr* mutant, all fractions showed great reduction compared to wt. Collectively, this shows that secretion of Ebba680 binding target is largely controlled by *agr*.

Whereas *agrA* controls the expression of PSM peptides, it is their fibrillated form that provides structural stability to the biofilm. To investigate binding of Ebba680 to fibrillated PSMs (fPSMs), we used synthetic fibrillated PSMα1-4, which were pre-stained with Thioflavin T (ThT) to confirm the fibrillated state. When detecting ThT fluorescence, highest intensity was observed in fPSMα2 and fPSMα3, followed by fPSMα1, whereas no signal was observed from fPSMα4 (Fig. 4F). Increasing the concentration of Ebba680 led to decreased ThT intensity, confirming that Ebba680 binds to the PSM fibril. Whether Ebba680 displaces ThT, or if the observed fluorescence occurs because of fluorescence resonance energy transfer (FRET) between ThT and Ebba680 located in close spatial proximity to each other needs further investigation. When detecting Ebba680 fluorescence in the same samples, we observed increased intensities with α2 > α1 > α3 > α4 (Fig. 4G), confirming that binding of Ebba680 to fPSMs leads to increased fluorescence intensities. Finally, we analysed whether binding of Ebba680 is restricted to the aggregated form of PSMs. In fibrillation kinetic studies using PSMβ1, low intensity signals at start of the fibrillation process indicated no binding to the non-aggregated form, whereas increased Ebba680 fluorescence intensity was observed as fibrillation occurred, confirmed by ThT (Fig. 4H). Binding of Ebba680 to fPSMs but not the non-fibrillated peptides may explain the delayed appearance of Ebba680 fluorescence in the growing biofilms.

### 2.5 Tracking of ECM production adds a new dimension to colony growth dynamics quantification

**Fig. 5:**
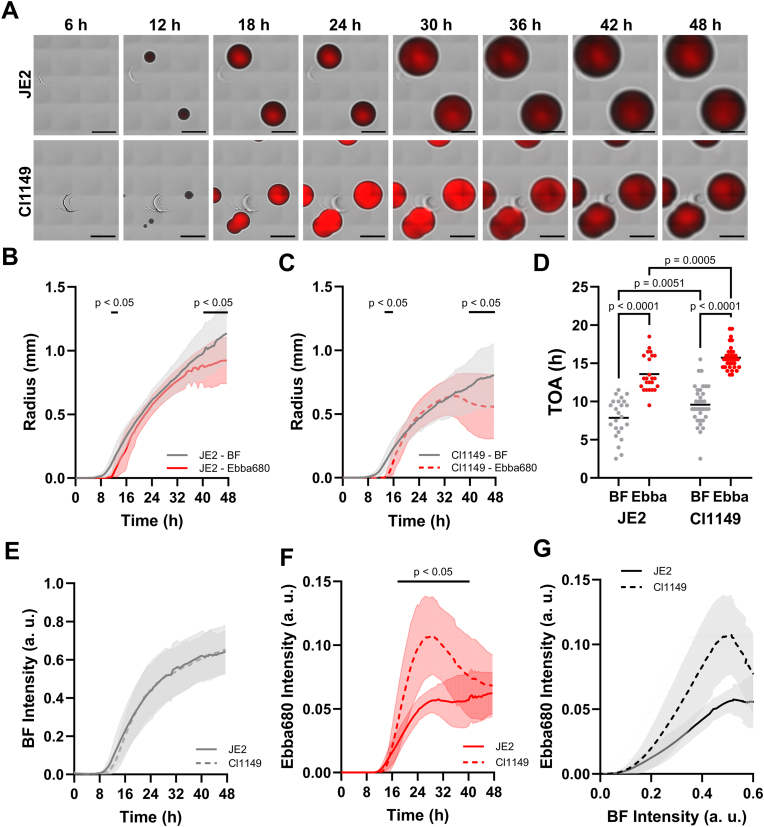
**Colony growth dynamics quantification of JE2 and Cl1149 A)** Growth kinetics of single cell derived colonies of *S. aureus* strains JE2 (upper) and Cl1149 (lower) grown on TSA + Ebba680 at 37 °C in an automated microscope. Overlays of images collected in the brightfield (grey) and Ebba680 fluorescence (red) channels are shown for one representative experiment (n = 3). Scale bar = 1 mm. **B–C)** Radius shown as mean value ± SD (shading) of **B)** JE2 and **C)** CI1149 colonies determined based on brightfield (BF) and Ebba680 fluorescence. Black squares above curves indicate timepoints where the difference between the means was significant with p > 0.05 determined by a two-way Anova test with Šídák's multiple comparisons test. **D)** The time of appearance (TOA) when radius >0 μm for JE2 and Cl1149 colonies determined based on brightfield (BF) and Ebba680 fluorescence (n = 3). P-values of comparisons between all groups were obtained using a Two-way Anova test with uncorrected Fisher's LSD test for multiple comparisons. **E-F)** Intensity shown as mean value ± SD (shading) of JE2 and CI1149 colonies in **E)** Brightfield (BF) and **F)** Ebba680 channels. Black squares above curves indicate timepoints where the difference between the means was significant with p > 0.05 determined by a two-way Anova test with Šídák's multiple comparisons test. **G)** Correlation between brightfield (BF) intensity and Ebba680 intensity for JE2 and Cl1149 colonies. (For interpretation of the references to colour in this figure legend, the reader is referred to the Web version of this article.)

Tracking of bacterially produced extracellular components may aid the phenotypic evaluation of clinical isolates. We therefore assessed if Ebba680 is compatible with Colony Time-lapse application (ColTapp) [66] for extended colony growth dynamics quantification and real-time measurement of extracellular component secretion within the biomass. We used the JE2 strain from the USA300 lineage of community-associated methicillin-resistant *S. aureus* and Cl1149, a clinical *S. aureus* isolate, as both have been previously characterised using ColTapp [63,66]. Colonies of JE2 and Cl1149 were grown on TSA + Ebba680 for 48 h and imaged every 30 min. While colony radius and intensity were determined based on brightfield images, Ebba680 fluorescence was used to determine the radius and intensity of secreted extracellular components. As colonies grew, Ebba680 fluorescence appeared with a delay in both strains. In JE2, Ebba680 fluorescence was consistently strong in the centre and fading towards the edges, while Cl1149 colonies showed strong Ebba680 staining of the entire colony for approximately 30 h before the pattern shifted and became more like in JE2 (Fig. 5A). Colony radius analysis showed JE2 colonies to reach a maximum radius of 1.14 ± 0.24 mm while Cl1149 colonies were smaller with a maximum radius of 0.81 ± 0.25 mm (Fig. 5B and C). This difference likely originated from differences in lag times and growth rates. By measuring the TOA as soon as a radius was detected by ColTapp, JE2 showed an average TOA of 7.85 ± 2.58 h compared to 9.57 ± 2.59 h for Cl1149 (Fig. 5D). In the time interval 12–24 h, JE2 had a higher growth rate (JE2_slope_ = 28.18 ± 0.18 μm/h, R^2^ = 0.8930) compared to Cl1149 (Cl1149_slope_ = 20.87 ± 0.18 μm/h, R^2^ = 0.8064) confirming that the larger size of JE2 colonies may be explained by a shorter lag phase and faster growth rate. The radii of extracellular secreted components based on Ebba680 fluorescence aligned well with colony radii during most time of incubation, however, significant differences were observed in between 11.5 and 13 h and after 40.5 h for JE2 (Fig. 5B) and between 13.5 and 15.5 h and after 40 h in Cl1149 (Fig. 5C). The end of the earlier time windows overlapped with the average TOA of Ebba680 fluorescence radii, which for JE2 was 13.58 ± 2.31 h and for Cl1449 15.71 ± 1.53 h (Fig. 5D). Compared to the TOA of colony radii, Ebba680 radii are detected with a delay of 5.73 ± 3.46 h in JE2 and 6.14 ± 3.00 h in Cl1149 colonies (Supplementary Table 2). With a z-score of z = 1.655, the difference between the delays is not considered significant with a 95 % confidence level indicating that in colonies of both strains, it takes approximately the same time to synthesise and secrete the extracellular components to which Ebba680 binds. The disconnect between colony- and Ebba680 fluorescence radii at later time leads to a radius of Ebba680 fluorescence which is 0.21 ± 0.30 mm smaller than JE2 colonies and 0.25 ± 0.35 mm smaller than Cl1149 colonies at the end of the incubation period. The relationship between colony- and Ebba680 fluorescence radii during growth suggested that the expression of the extracellular component(s) to which Ebba680 binds is growth dependent. Although the importance of such information is currently unclear, it may be of importance for future classification of clinical strains.

Besides threshold-based radius quantification, ColTapp allows intensity measurement within the colony area. Brightfield intensities of the two strains overlapped (Fig. 5E). Major differences were observed however in the Ebba680 fluorescence intensity (Fig. 5F). While the Ebba680 intensity in Cl1149 colonies was higher, both strains reached a peak intensity at around 30 h (JE2_tpeak_ = 30.59 ± 4.04 h and Cl1149_tpeak_ = 28.93 ± 3.96 h, Unpaired *t*-test shows no significant difference with p = 0.0859). Between 16 and 24 h, the intensity increase was twice as much in Cl1149 compared to JE2 (Cl1149_slope_ = 9.97 ± 0.22 ma.u./h, R^2^ = 0.9975 and JE2_slope_ = 4.11 ± 0.053 ma.u./h, R^2^ = 0.9929). This resulted in a circa 2-fold higher peak intensity of Cl1149 compared to JE2 (Cl1149_Ipeak_ = 0.12 ± 0.03 h and JE2_Ipeak_ = 0.06 ± 0.01 h, Unpaired *t*-test shows significant difference with p < 0.0001). Thus, bacterial expression and secretion of component(s) acting as extracellular target for Ebba680 occurs at a much higher rate in Cl1149 compared to JE2 during this growth phase. From 30 h onwards, the fluorescence intensity of JE2 colonies entered a plateau, while Cl1149 showed an intensity decrease. To understand the correlation between biomass growth, reflected by brightfield intensity, and production of extracellular Ebba680 binding targets, reflected by Ebba680 fluorescence, we plotted the intensities of Ebba680 versus Brightfield (BF) at each time point throughout incubation (Fig. 5G). Both strains showed an almost immediate increase of biomass, while production of extracellular Ebba680 binding targets was delayed. However, once production started, the amount of Ebba680 binding targets increased proportionally to the biomass. When biomass reached about 80 % of its maximum, ECM production levelled off in JE2 colonies and decreased in Cl1149 colonies. This shift may involve quorum sensing regulatory mechanisms or altered nutrient conditions. Whether the stark decrease in ECM production at higher biomass in Cl1149 colonies was caused by active biological events such as degradation or remodelling of extracellular matrix components, or photophysical issues such as quenching is currently unknown. Taken together, this illustrates that tracking of extracellular components in colonies with Ebba680 provides a novel opportunity to extract and visualize strain dependent differences unachieved by previous methods.

## 3 Discussion

Colony phenotypes serve as useful indicators of biofilm-associated traits. The agar-based assay reported here is first-of-its-kind enabling real-time studies of biofilm formation and spatial-temporal localization of extracellular matrix components in gram-positive bacterial colonies. Essential to our assay is the non-toxic optotracer EbbaBiolight 680, present in the agar on which the biofilms form. In contrast to conventional fluorophores, the fluorescent signal from Ebba680 remains OFF until binding targets are produced and become accessible. As target-binding switches Ebba680 fluorescence ON, dynamic studies can be performed in which the optotracer reports the production of ECM and the growth of the biofilm in real-time. We show that the assay works equally well, albeit with different kinetics, for drop-inoculated biofilm macrocolonies as for single cell derived biofilm colonies.

In Gram-negative bacteria, the rdar (red, dry and rough) morphotype is a key phenotype associated with biofilm formation, reflecting production of ECM components including the functional amyloid curli and cellulose. The rdar morphotypes are usually visible after 2–5 days incubation on Congo red-containing agar plates [51,53], whereas Ebba680 enables detection of amyloid curli within 24 h in Salmonella and UPEC macrocolonies [37,54,55]. A significantly shortened read-out time is thus achieved by Ebba680 compared to traditional methods. As short timeframes are essential in clinical diagnostics, the present study focused on early detection in the range of 0–48 h of ECM formation in *S. aureus* colony biofilms. The 48 h timepoint was also identified as a technical limit to the method, since tracking of growth dynamics is obstructed when single cell derived colonies start to grow out of the field-of-view.

The extracellular location and delayed appearance of Ebba680 fluorescence in *S. aureus* colonies led to the hypothesis that fPSMs, also amyloids and previously observed in biofilm forming strains of *S. aureus* USA300 [23,67], may be a Ebba680 binding target. To investigate this, we used ThT as a conventional dye for staining functional amyloids, together with Ebba680. The fluorophore concentrations and acquisition settings were chosen according to supplier's instructions to achieve optimal signal-to-noise ratios for each dye. Consequently, Ebba680 was used at much lower concentration compared to ThT. The lower brightness of the red/far-red Ebba680 compared to the blue/green ThT is a common effect, reflecting a combination of lower quantum yields and intrinsically lower photon energy of red emission, as well as reduced detector sensitivity at longer wavelengths [[68], [69]]. We confirmed binding of Ebba680 to aggregated forms of synthetic PSMα1-4, with the observed differences in signal intensities likely resulting from variations in the fibril structures, as shown for PSMα fibrils [27,30,70]. Ebba680 binds promiscuously, evidenced by its ability to also bind cellulose in Salmonella biofilms [39,71] as well as β-glucans in *Candida albicans* and similar molecules have been shown to fluoresce upon binding to peptidoglycan and teichoic acids [64,65]. Here, we identify fPSMs as an important but not unique binding target for Ebba680, other possible candidates include the PIA polysaccharide and the Bap functional amyloid.

The role of PSMs in staphylococcal biofilm formation and function is well described [29,72,73], but their localization throughout the biofilm has remained elusive. Direct visualization of PSMs and their fibrillated forms in the biofilm matrix has been challenging to address due to their small size and dynamic nature. The new labelling technique described here demonstrates fPSMs to be partly interspersed between cells, as well as forming a cap-like structure on the outer surface of mature *S. aureus* biofilm colonies. This fits with spatial data obtained from other biofilm forming gram-negative bacteria. In UPEC, Ebba680 revealed the involvement of curli in ECM microstructure formation, which conveyed significant hydrophobicity to the biofilm [55]. It may be that the cap-like structure on the outer surface observed in this work may modulate the physical properties of the *S. aureus* biofilm. This advance will allow for extensive new molecular studies of the spatial, temporal and functional role of fPSMs in gram-positive biofilms, a critical element in antibiotic resistance and infection outcome.

In Gram-positive bacteria, tracking of colony growth dynamics provides a valuable tool for phenotypical characterization of single cell derived colonies [66,74]. Here, we showed how quantification of real-time ECM staining can be used to complement colony growth dynamics as a tool to understand if changes in colony growth rate are related to biofilm formation. Comparing a well-defined USA300 MRSA strain with a clinical isolate from a biofilm-associated infection revealed striking differences in temporal expression and signal intensity, indicating altered expression kinetics of ECM components such as the fibrillated PSMs. Further work is needed to define the molecular mechanisms of these differences, but the approach described can help identify strong biofilm forming strains with spatial and temporal resolution. The ability to rapidly screen ECM formation in single cell colonies on agar plates will streamline not only the basic research into biofilm development but can also contribute to the classification of clinical isolates. This approach can facilitate direct analysis of phenotypic heterogeneity within populations recovered from infected materials and help guide treatment decisions.

## 4 Methods

### 4.1 Bacterial strains, media, and supplements

Bacterial strains and plasmids are listed in Supplementary Table 1. Stable expression of GFP under the control of the sarA P1 promoter was achieved by transforming SH1000 with plasmid pSGFPS1 (BEI Resources, NIAID, NIH) as described [75], thereby generating SH1000-pSGFPS1 (SH1000-GFP). Tryptic soy broth (TSB), tryptic soy agar (TSA) and Trimethoprim (used at 10 μg/mL) were from Sigma-Aldrich (Sweden). The optotracer EbbaBiolight 680 (Ebba680) from Ebba Biotech AB (Sweden) was used at 2 μg/mL when supplementing liquid (TSB) and agar based (TSA) cultures.

### 4.2 *S. aureus* growth in liquid culture

Overnight cultures were prepared by inoculating 5 mL TSB with the selected strain and incubated with agitation at 37 °C overnight. Exponential phase cultures were prepared from overnight cultures diluted 1:100 in 10 mL TSB and incubated with agitation at 37 °C until OD_600_ reached 0.3 (∼10^8^ CFU/mL). SH1000-GFP was routinely cultured in the presence of Trimethoprim.

For kinetic recordings of liquid cultures in 96-well plates, overnight cultures were diluted 1:100 in TSB with or without Ebba680 (2 μg/mL) from which 200 μL was added to each well of a transparent flat bottom 96-well plate (Sarstedt, Sweden). The plate was incubated in a microplate spectrophotometer (Infinite® M1000, Tecan, Switzerland) at 37 °C for 20 h under static conditions. Absorbance at 600 nm and fluorescence with λ_Ex_ = 530 nm and λ_Em_ = 650 ± 20 nm were recorded at 15 min intervals throughout incubation.

### 4.3 6-well plate biofilm assay

Plates were prepared by adding TSA (2 mL) with or without Ebba680 (2 μg/mL) to the wells of a 6-well plate (Sarstedt, Germany). For growth of drop-inoculated macrocolonies, 10 μl of an exponential phase TSB culture or control TSB medium was placed centrally on the agar of each well. For growth of single cell derived colonies, the exponential phase culture was diluted 1:10^5^ in TSB and 10 μl of the inoculate or control TSB medium was placed centrally on the agar together with 150 μl TSB. The liquid was immediately spread across the agar surface using autoclaved glass beads to ensure even distribution of bacteria. Plates were incubated at 37 °C for 24 h either in a plate reader or an automated microscope depending on the experiment.

When biofilm formation was analysed by ColTapp tracking, single colonies grown overnight on Columbia Sheep Blood agar plates (CSB, BioMérieux, Switzerland) were resuspended in 200 μl Phosphate Buffered Saline (PBS). Following serial dilution, 5 μl drops were added onto the agar in a 3x3 grid. The plates were incubated at 37 °C for 48 h in a fully automated inverted microscope.

### 4.4 Automated microscopy

Imaging of SH1000 and SH1001 in 6-well plates was achieved using a Lionheart™ FX Automated Microscope (Ramcon, Sweden) with Gen5 software for imaging and microscopy (Ramcon, Sweden). A1.25X Plan Apochromate WD5 NA 0.04 objective was used to acquire brightfield and fluorescence images. To detect Ebba680 fluorescence a propidium iodide filter cube was used with 523 nm LED excitation. The LED intensity, integration time, and gain settings were 5, 100 μs, 0 for brightfield and 10, 200 μs, 20 for Ebba680 detection. All images were acquired in ‘Tiling’ mode wherein a matrix of 8x5 images was collected and then stitched together. The brightfield images were used as reference for stitching to visualize a whole well. For kinetic monitoring of biofilm growth, the automated microscope was programmed to maintain a constant temperature of 37 °C and image for 24 h at 30 min intervals. After the experiment, Movies were generated by integrating the images over 24 h, and raw images were exported for each timepoint and channel.

### 4.5 Area scan using fluorescence plate reader

To scan the entire well, a plate reader (Infinite® M1000, Tecan, Switzerland) was programmed to scan 15x15 area units with 2.5 mm diameter each. Fluorescence was read from the bottom with λ_Ex_ = 530 nm and λ_Em_ = 650 ± 10 nm. For kinetic imaging, the plate reader was programmed to maintain a constant temperature of 37 °C and to perform a reading every 15 min for 24 h.

### 4.6 Confocal imaging

After 24 h incubation, the agar – along with the colony – was carefully isolated from the well and placed upside down in a 35 mm Ibidi μ-Dish 35 with glass bottom for imaging (Ibidi, Germany). The samples were imaged using a Zeiss LSM 900 Airyscan Confocal Microscope and Zen software (Zeiss, Germany) with a Plan Apochromat 63X/1.4 DIC ∞/0.17, WD 0.19 mm oil immersion objective. Airyscan mode was used as indicated to achieve additional 6X digital zoom. Ebba680 fluorescence was detected using the PI channel with 0.4 % laser power at λ_Ex_ = 561 nm, 850 V master gain, and 1.0 digital gain. GFP fluorescence from GFP expressing bacteria was detected using the EGFP channel with 0.4 % laser power at λ_Ex_ = 488 nm, 850 V master gain, and 1.0 digital gain. Images were captured as z-stacks with 0.15 μm step size between different z-slices. Image stacks were exported as czi files and Airyscan stacks were exported after “Airyscan Processing” using Zen Software (Zeiss, Germany).

### 4.7 Colony Time-lapse application (ColTapp) for tracking colony growth of JE2 and Cl1149

A fully automated Olympus IX83 P2ZF inverted microscope controlled via CellSense 3.2 (Olympus) was used for imaging of the 6-well plate. Brightfield (10 ms exposure, 60 intensity IX3 LED) and Ebba680 fluorescence (50 ms exposure, 30 % intensity, 525 nm CoolLED pE-4000, quad-band filter for DAPI/GFP/CY3/CY5) images were captured at 16-bit resolution in 30 min intervals using a 10x air objective (Olympus) and an ORCA-flash 4.0 sCMOS camera (Hamamatsu). CellSense and inbuilt automatic stitching was used to acquire a large area containing all nine drops. The temperature was constantly maintained at 37 °C.

A custom MATLAB (Mathworks) script was used to select the area of each drop with intended colony density and to extract the brightfield and Ebba680 channels into separate.tif files (https://github.com/ColTapp/auxillary/releases/tag/v1.0). The previously published ColTapp was modified (https://github.com/ColTapp/matlab-code/releases/tag/v1.2) to be able to read in multichannel images. ColTapp was used to generate multichannel intensity kymographs of each colony, from which the radius and normalized intensity were determined.

### 4.8 Supernatant and pellet analysis

Overnight cultures were prepared and divided in two parts. One part was left untreated and stored on ice, the other was centrifuged at 12000 rpm for 10 min at room temperature. After centrifugation, supernatants were carefully separated from cell pellets. The supernatant was stored on ice. The cell pellet was resuspended in TSB to the original volume and then stored on ice. Ebba680 (2 μL/mL) was added to all samples and incubated for 45 min at room temperature. TSB + Ebba680 (2 μL/mL) was used as blank. Absorbance at 600 nm and fluorescence with λ_Ex_ = 530 nm and λ_Em_ = 650 ± 20 nm of samples and blank was recorded using a plate reader (Infinite® M1000, Tecan, Switzerland).

### 4.9 PSM analysis

Synthetic N-terminally formylated PSM peptides (0.5 mg/mL) with >95 % purity (GenScript, Netherlands) were pre-treated, aggregated, and stained with 40 μM Thioflavin T (ThT) as described and then frozen until further use[27,27,76,76]. The pre-stained aggregates were thawed, diluted in ultrapure water to 0.1 mg/ml and incubated with 0, 2 or 7 μg/mL Ebba680 for 10 min. Emission of ThT-stained aggregates was recorded at λ_Em_ = 490 ± 10 nm with λ_Ex_ = 450 nm. Emission of Ebba680-stained aggregates was recorded at λ_Em_ = 650 ± 10 nm with λ_Ex_ = 540 nm. To determine the aggregation kinetic of PSM β1, dry lyophilized peptides (GenScript, Netherlands) were suspended in individual Eppendorf tubes at a 1:1 concentration of 0.5 mg/ml in hexaflouroisopropanol (Sigma Aldrich, Denmark) and Trifluoroacetic acid (Sigma Aldrich, Denmark) and kept on ice. Each tube was sonicated 5 × 20 s (Qsonica model CL-334 probe sonicator, Qsonica, United States) and incubated for 1 h in room temperature. Tubes were then placed in a chemical hood to evaporate overnight. The aliquots were further evaporated using a Speedvac at 1000 rpm for 3 h, then suspended in DMSO and spun down at 10 000 rpm for 5 min and diluted to 0.1 mg/ml in ultrapure water containing 2 μg/mL Ebba680 or 64 μg/mL ThT (200 μM). Kinetic analysis was conducted on a plate reader (FLUOstar Omega, BMG Labtech, Germany). Using bottom read mode, Ebba680 fluorescence was detected with λ_Ex_ = 544 nm and λ_Em_ = 635 nm and ThT fluorescence with λ_Ex_ = 450 nm and λ_Em_ = 480 nm every 5 min for 38 h with shaking at 200 rpm for 20 s prior to measurement.

### 4.10 Data analysis and statistics

ImageJ 1.54f (NIH, USA) was used to import single images from the automated microscope into stacks with 16-bit images. Timelapse images were imported as image sequences, and the “Bio-formats” plugin was used to import czi files into z-stacks. The “Measure Stack” function was used on timelapse sequences and on z-stacks to determine the average fluorescence intensity or brightness of each image in the sequence or stack. The “Orthogonal views” function was used to create orthogonal projections of the stacks and “3D Viewer” was used to render 3D reconstructions of stacks. The “Analyze Particles” function was used to determine the radius of the macrocolonies with size: 50000–200000 px^2^ and circularity: 0.0–1.0 on auto-thresholded images. Post-processing of data was performed using Python 3 with packages pandas and numpy (https://github.com/AIMES-ARD/StaphProject). GraphPad Prism 10 (GraphPad Software, USA) was used for plotting and statistical analysis. Normalization of Intensities (I) was performed as Min-Max normalization (I_max_ = 1, I_min_ = 0) or normalization against the maximum (I_max_ = 1) in each replicate.

## CRediT authorship contribution statement

**Tianqi Zhang:** Methodology, Investigation. **Julian Bär:** Writing – review & editing, Methodology, Investigation, Formal analysis, Data curation. **Lovisa Risberg:** Investigation. **Alejandro Gómez Mejia:** Writing – review & editing, Investigation. **Hugo Hammar:** Investigation. **Susanne Löffler:** Writing – review & editing, Writing – original draft, Visualization, Supervision, Project administration, Formal analysis, Data curation, Conceptualization. **Daniel Erik Otzen:** Writing – review & editing, Methodology. **Maria Andreasen:** Writing – review & editing, Resources, Methodology. **Rikke Louise Meyer:** Writing – review & editing, Conceptualization. **Keira Melican:** Writing – review & editing, Writing – original draft, Supervision, Conceptualization. **Annelies S. Zinkernagel:** Writing – review & editing, Supervision, Resources, Funding acquisition, Conceptualization. **Agneta Richter-Dahlfors:** Writing – review & editing, Writing – original draft, Supervision, Resources, Project administration, Methodology, Funding acquisition, Conceptualization.

## Declaration of competing interest

The authors declare the following financial interests/personal relationships which may be considered as potential competing interests:Susanne Loffler reports a relationship with Ebba Biotech AB that includes: board membership and equity or stocks. Agneta Richter-Dahlfors has patent licensed to Ebba Biotech AB. Intellectual properties relevant to this work is licensed by Ebba Biotech AB from Richter Life Science Development AB, founded and co-owned by A. Richter-Dahlfors. Richter Life Science AB is major owner of Ebba Biotech AB, which commercializes optotracers for uses as described in this article. Given her role as member of the Editorial board of Biofilm, Rikke Louise Meyer had no involvement in the peer-review of this article and has no access to information regarding peer-review. Full responsibility for the editorial process for this article was delegated to another journal editor. If there are other authors, they declare that they have no known competing financial interests or personal relationships that could have appeared to influence the work reported in this paper.

## Data Availability

Data will be made available on request.
