# Supplementary material for: Dynamic visualization of extracellular matrix components in S. aureus colony biofilms reveals functional amyloids leading to the formation of cap-like structures
Source: Biofilm. 2025 Sep 18;10:100318. doi: 10.1016/j.bioflm.2025.100318 (PMC12510064; doi:10.1016/j.bioflm.2025.100318)
Supplement: Multimedia component 1 [file mmc1.docx]

# SUPPLEMENTARY INFORMATION

**Dynamic visualization of extracellular matrix components in *S. aureus* colony biofilms reveals functional amyloids leading to the formation of cap-like structures**

Tianqi Zhang^1,2,7^, Julian Bär^3,7^, Lovisa Risberg^1,2^, Alejandro Gómez Mejia^3^, Hugo Hammar^1,2^, Susanne Löffler^1,2,*^, Daniel Erik Otzen^4^, Maria Andreasen^5^, Rikke Louise Meyer^4^, Keira Melican^1,2,6^, Annelies S. Zinkernagel^3^, Agneta Richter-Dahlfors^1,2,*^

^1^ AIMES – Center for the Advancement of Integrated Medical and Engineering Sciences, Karolinska Institutet and KTH Royal Institute of Technology, SE-171 77, Stockholm, Sweden

^2^ Department of Neuroscience, Karolinska Institutet, SE-171 77, Stockholm, Sweden

^3^ Department of Infectious Diseases and Hospital Epidemiology, University Hospital Zurich, University of Zurich, Zurich, Switzerland

^4^ Interdisciplinary Nanoscience Centre (iNANO) and Department of Molecular Biology and Genetics, Aarhus University, Aarhus 8000, Denmark

^5^ Department of Biomedicine, Aarhus University, Aarhus 8000, Denmark

^6^ Department of Medicine Solna, Karolinska Institutet, Stockholm, Sweden

^7^ These authors contributed equally

* Corresponding authors: agneta.richter.dahlfors@ki.se, susanne.loffler@ki.se

# Page Index Page

Supplementary Table 1 3

Supplementary Table 2 4

Supplementary Figure 1 5

Supplementary Figure 2 6

Supplementary Figure 3 7

Supplementary Figure 4 8

Supplementary Figure 5 9

Supplementary Figure 6 10

Supplementary Figure 7 11

Supplementary Figure 8 12

Legends to Supplementary Movies 1-9 13

**Supplementary Table 1** Strains and plasmids

| **Strains** | **Characteristics** | **Source** |
| --- | --- | --- |
| SH1000  (ARD184) | Wild-type *S. aureus* derived from 8325-4 lineage | BEI Resources, NIAID, NIH: Staphylococcus aureus, Strain SH1000, NR-55396 |
| SH1001  (ARD183) | SH1000 *agr*::tet | Ref. 1 |
| JE2 | JE2 is a methicillin-resistant *S. aureus* (MRSA) strain and is a USA300 isolate | Network on Antimicrobial Resistance in Staphylococcus aureus (NARSA) for distribution by BEI Resources, NIAID, NIH: *S.aureus* subsp. *aureus*, Strain JE2, NR-46543. |
| CI-1149 | *S. aureus* patient-derived isolate | Ref. 2 |
| SH1000-GFP  (ARD340) | GFP-labeled SH1000 transformed with plasmid pSGFPS1 | Ref. 3 |
|  |  |  |
| **Plasmids** | **Characteristics** | **Source** |
| pSGFPS1 | Vector for green fluorescent protein expression and trimethoprim resistance (Tmp^R^) | BEI Resources, NIAID, NIH: *Staphylococcus aureus* Fluorescent Reporter Plasmid pSGFPS1, Recombinant in *Staphylococcus aureus*, NR-51163 |

**References**

1. Horsburgh, M. J. *et al.* δb modulates virulence determinant expression and stress resistance: Characterization of a functional rsbU strain derived from *Staphylococcus aureus* 8325-4. *J Bacteriol* **184**, 5457–5467 (2002).

2. Huemer, M. *et al.* Molecular reprogramming and phenotype switching in *Staphylococcus aureus* lead to high antibiotic persistence and affect therapy success. *Proc Natl Acad Sci U S A* **118**, e2014920118 (2021).

3. Rodriguez, M. D., *et al*. Construction of Stable Fluorescent Reporter Plasmids for Use in *Staphylococcus aureus*. *Front Microbiol* **8**, 2491 (2017).

**Supplementary Table 2** Time of Appearance (TOA)

|  | TOA (h)  Brightfield | TOA (h)  Ebba680 fluorescence | Delay (h) |
| --- | --- | --- | --- |
| **Macrocolony**  SH1000 Image analysis | 4.33 ± 0.29 | 6.00 ± 0.00 | 1.67 ± 0.29 |
| **Single cell derived colony**  SH1000 Area Scan  JE2 ColTapp Radius  Cl1149 ColTapp Radius | n.a.*  7.85 ± 2.58  9.57 ± 2.59 | 10.38 ± 1.21  13.58 ± 2.31  15.71 ± 1.53 | n.a.*  5.73 ± 3.46  6.14 ± 3.00 |

* n.a. = not applicable

**Supplementary Figure 1**


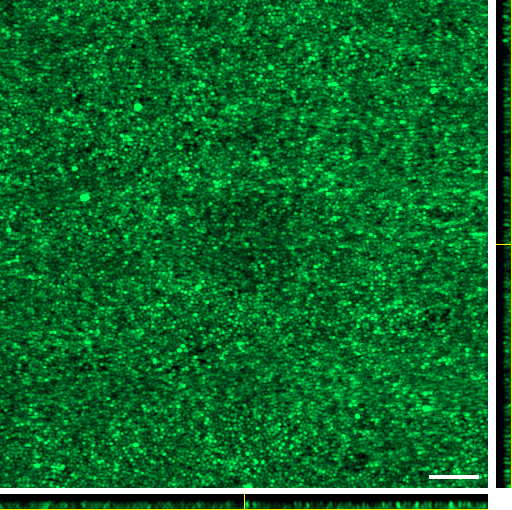


**Maximum intensity projection with orthogonal projections of SH1000-GFP macrocolonies grown on TSA for 24 h.** A confocal stack was acquired by CLSM using a 63X objective lens. The green channel (GFP) is overlayed with the red channel (Ebba680) as control. Scale bar = 10 µm.

**Supplementary Figure 2**


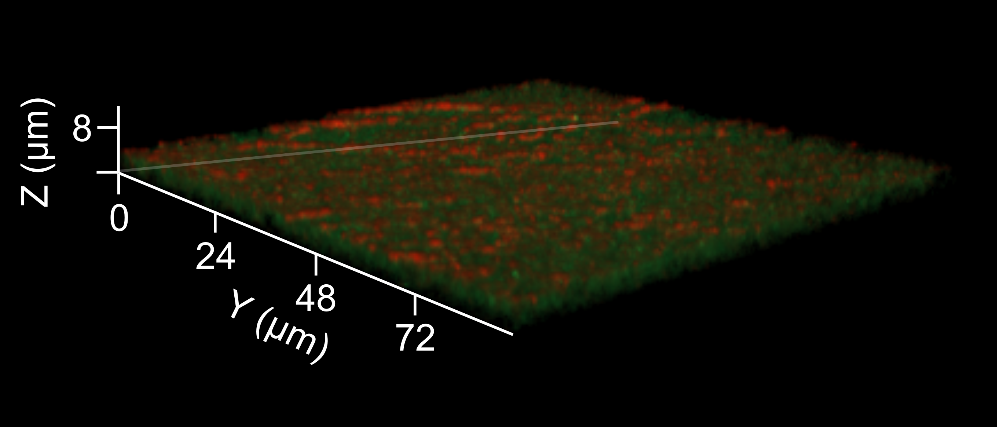


**A 3D reconstruction of a SH1000-GFP macrocolony grown on TSA+Ebba680 for 24h.** The confocal stack was acquired by CLSM using a 63X objective. Ebba680 fluorescence (red) is overlayed with fluorescence from GFP expressing cells (green). This image is associated with Figure 2A, Figure 2D, and Supplementary Movie 3.

**Supplementary Figure 3**

**
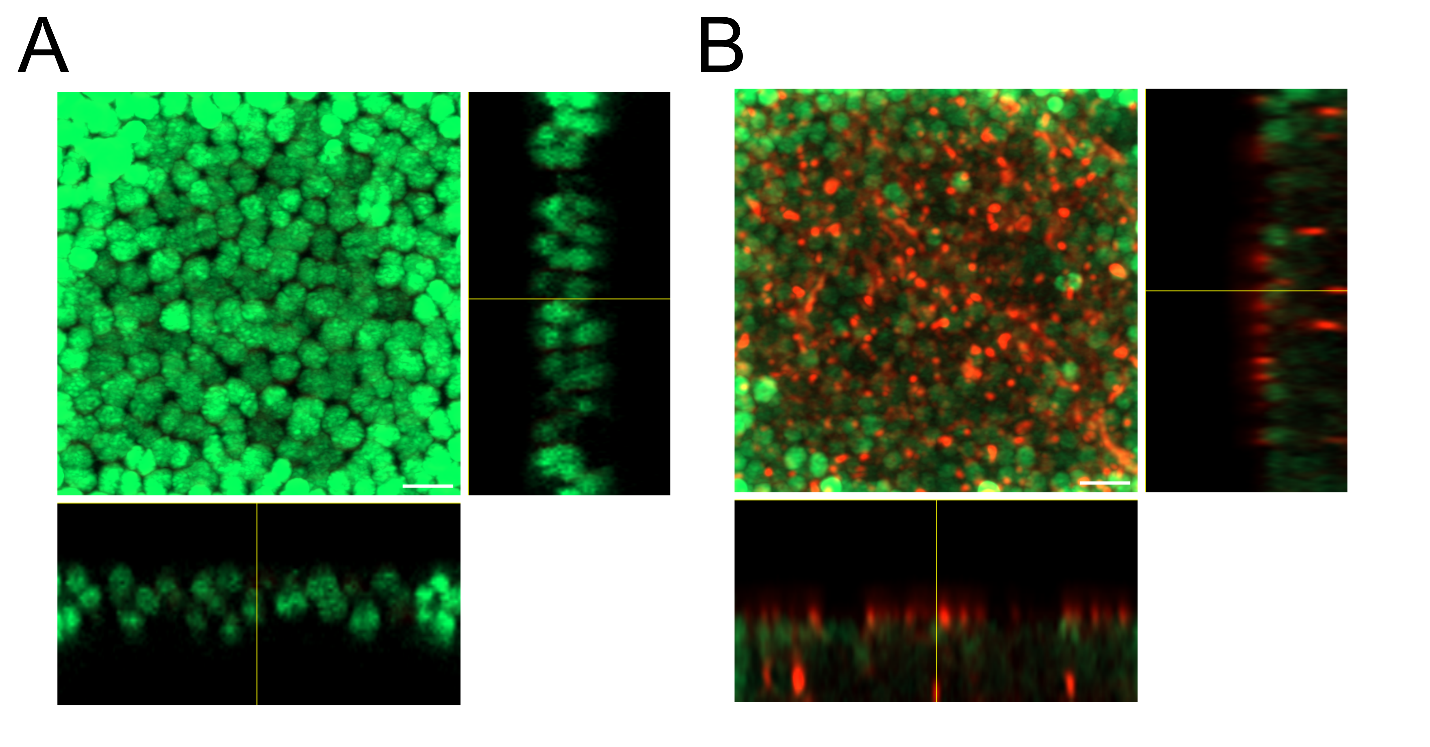
**

**Maximum intensity projections with orthogonal projections of SH1000-GFP macrocolonies grown on TSA+Ebba680.** **A**) 3 h and **B**) 6 h. Confocal stacks were acquired by CLSM using a 63X objective lens with 6x digital zoom and airyscan detection. Ebba680 fluorescence (red) is overlayed with fluorescence from GFP expressing cells (green). Scale bar = 2 µm.

**Supplementary Figure 4**

**
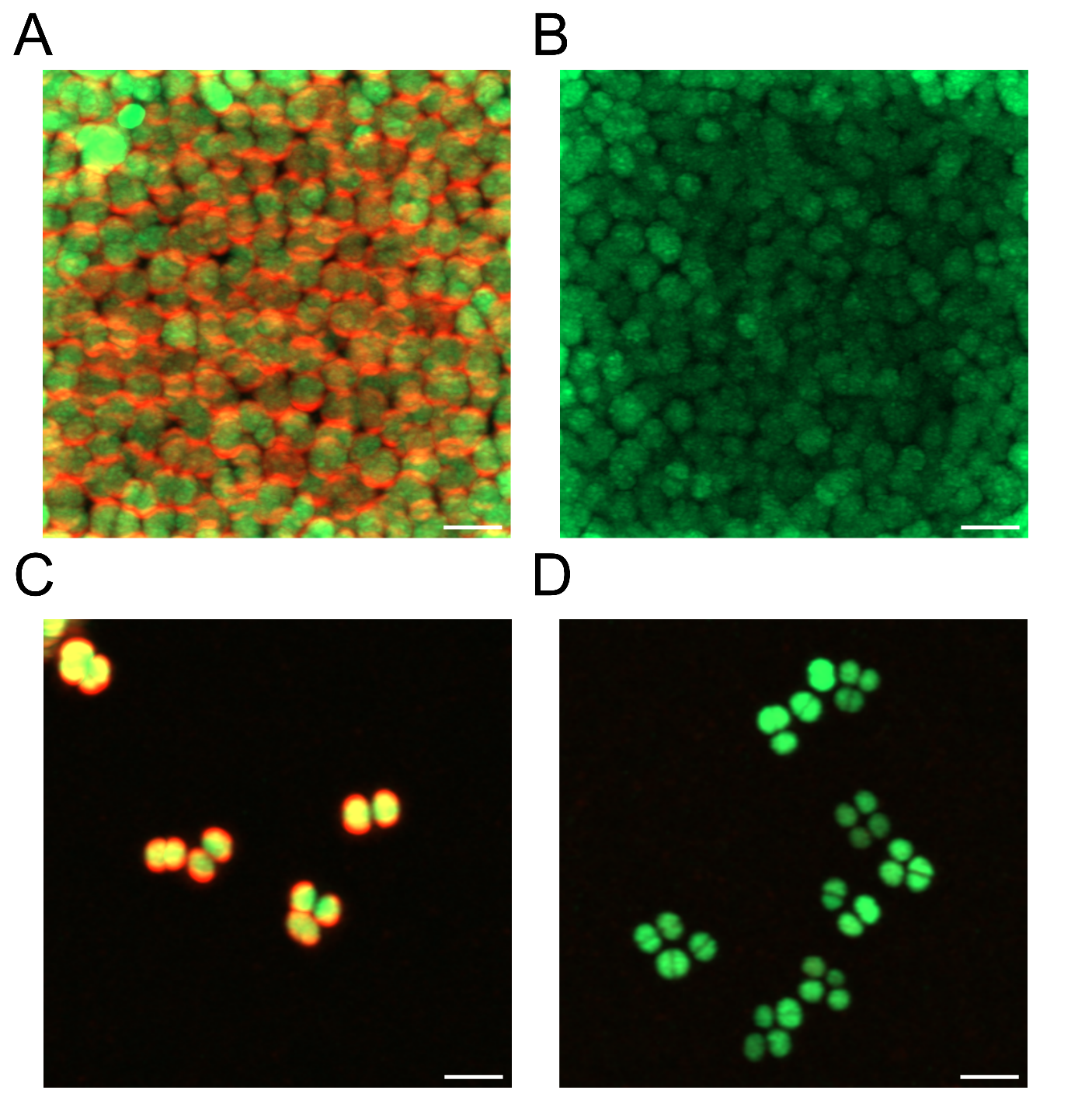
**

**SH1000-GFP grown at different conditions.** (**A, B**) Maximum intensity projections of SH1000-GFP macrocolonies grown for 3 h on **A**) TSA+Ebba680, and **B**) TSA, using 7X increased brightness in the red channel compared to standard settings. (**C, D**) Maximum intensity projections of SH1000-GFP harvested at early exponential phase of liquid culture growth in **A**) TSB+Ebba680, **B**) TSB, using 19X increased brightness in the red channel compared to standard settings. Confocal stacks were acquired by CLSM using a 63X objective lens with 6x digital zoom and airyscan detection. Ebba680 fluorescence (red) is overlayed with fluorescence from GFP expressing cells (green). Scale bar = 2 µm.

**Supplementary Figure 5**

**
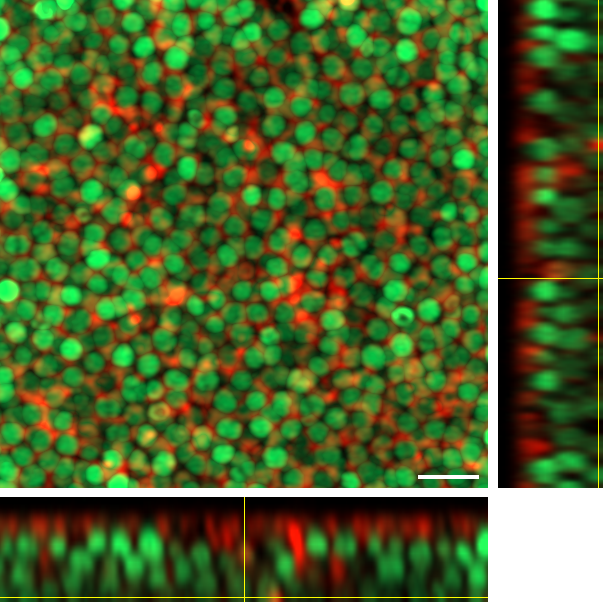
**

**Maximum intensity projection with orthogonal projections of a SH1000-GFP macrocolony grown on TSA+Ebba680 for 24 h.** Confocal stack acquired by CLSM using a 63X objective lens with 6x digital zoom and airyscan detection. Ebba680 fluorescence (red) is overlayed with fluorescence from GFP expressing cells (green). Scale bar = 2 µm.

**Supplementary Figure 6**

**
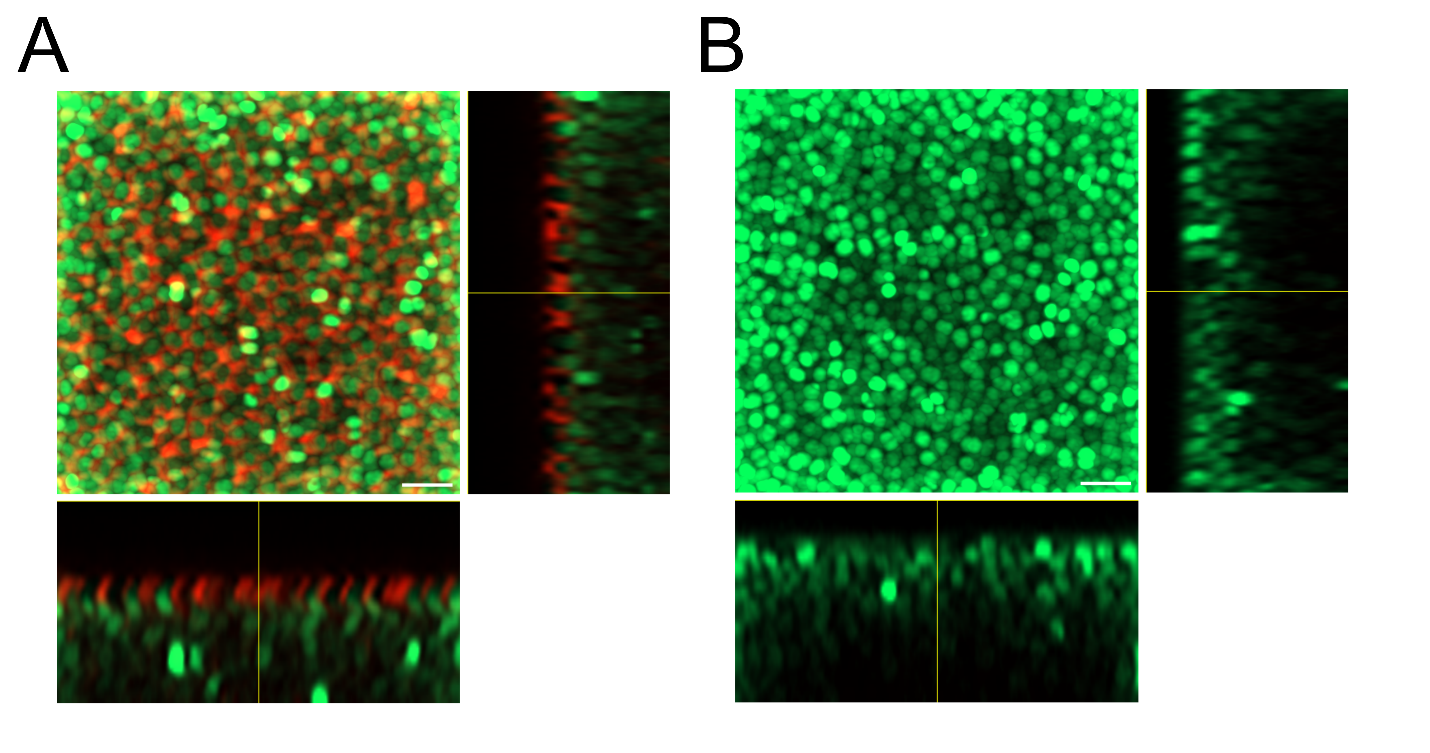
**

**Maximum intensity projections with orthogonal projections of SH1000-GFP single cell derived colonies grown for 24 h.** **A)** TSA+Ebba680, and **B)** TSA. Confocal stack acquired by CLSM using a 63X objective lens with 6X digital zoom and airyscan detection. Ebba680 fluorescence (red) is overlayed with fluorescence from GFP expressing cells (green). Scale bar = 2 µm.

**Supplementary Figure 7**

**
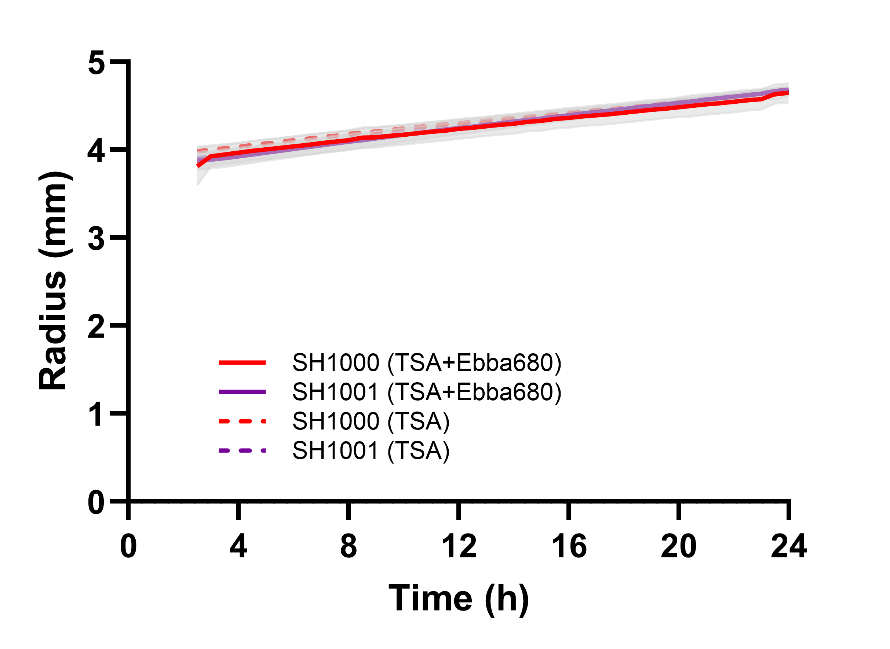
**

**Measurements of the radius of macrocolonies**. Macrocolonies formed by SH1000 (red) and SH1001 (black) macrocolonies during growth on TSA+Ebb680 (solid) and TSA (dotted). Data shown as mean values ± SD (grey shading).

**Supplementary Figure 8**

**
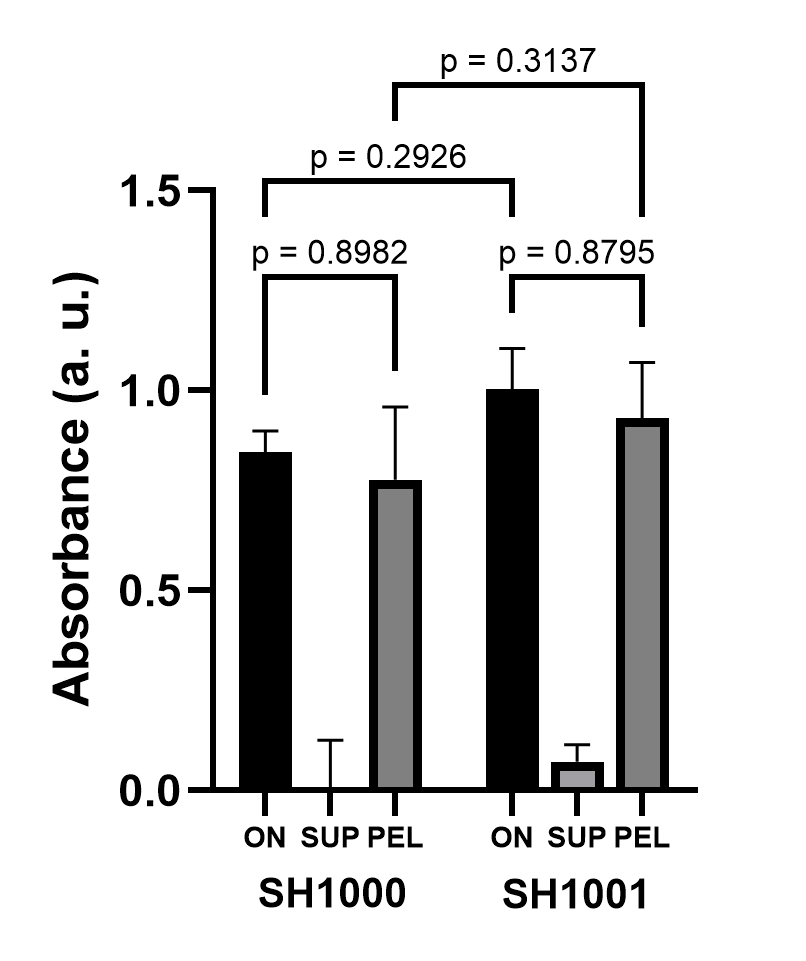
**

**Supernatant assay.** Absorbance at 600 nm of overnight cultures (ON), as well as the supernatant (SUP) and reconstituted pellet (PEL) of the overnight culture in TSB+Ebba680. Data are shown after baseline subtraction of the blank (TSB+Ebba680). A Two-way Anova with Šídák's multiple comparisons test between indicated groups shows that differences in mean values are not significant.

**Supplementary Movie 1 (mmc2.mp4)**

SH1000 macrocolony growing on TSA+Ebba680 for 24 h.

**Supplementary Movie 2 (mmc3.mp4)**

SH1000 growing on TSA for 24 h.

**Supplementary Movie 3 (mmc4.mp4)**

3D animation of confocal stack of SH1000-GFP growing on TSA+Ebba680 for 24 h. Movie acquired with 63X objective. Data shown as maximum intensity projection in Figure 2A.

**Supplementary Movie 4 (mmc5.mp4)**

3D animation of confocal stack of SH1000-GFP growing on TSA+Ebba680 for 3 h. Movie acquired with 63X objective and 6X digital zoom and airyscan detection. Brightness adjusted to match 6 h timepoint. Data shown as maximum intensity projection in Figure S3A.

**Supplementary Movie 5 (mmc6.mp4)**

3D animation of confocal stack of SH1000-GFP growing on TSA+Ebba680 for 6 h. Movie acquired using a 63X objective lens with 6X digital zoom and airyscan detection. Brightness auto-adjusted. Data shown as maximum intensity projection in Supplementary Figure S3B.

**Supplementary Movie 6 (mmc7.mp4)**

3D animation of confocal stack of SH1000-GFP macrocolonies growing on TSA+Ebba680 for 24 h. Movie acquired using a 63X objective lens with 6X digital zoom and airyscan detection. Data are shown as maximum intensity projection in Figure S5.

**Supplementary Movie 7 (mmc8.mp4)**

3D animation of confocal stack of SH1000-GFP colonies growing on TSA+Ebba680 for 24 h. Movie acquired with 63X objective and 6X digital and airyscan detection. Data are shown as maximum intensity projection in Figure S5.

**Supplementary Movie 8 (mmc9.mp4)**

SH1001 growing on TSA+Ebba680 for 24 h.

**Supplementary Movie 9 (mmc10.mp4)**

SH1001 growing on TSA for 24 h.
